# Transcranial Magnetic Stimulation Intensities in Cognitive Paradigms

**DOI:** 10.1371/journal.pone.0024836

**Published:** 2011-09-29

**Authors:** Jakob A. Kaminski, Franziska M. Korb, Arno Villringer, Derek V. M. Ott

**Affiliations:** 1 Department of Cognitive Neurology, Max Planck Institute for Human Cognitive and Brain Sciences, Leipzig, Germany; 2 Faculty of Medicine, University of Leipzig, Leipzig, Germany; 3 Center for Cognitive Neuroscience, Duke University, Durham, North Carolina, United States of America; 4 Department of Neurology, University Clinic Leipzig, Leipzig, Germany; City of Hope National Medical Center and Beckman Research Institute, United States of America

## Abstract

**Background:**

Transcranial magnetic stimulation (TMS) has become an important experimental tool for exploring the brain's functional anatomy. As TMS interferes with neural activity, the hypothetical function of the stimulated area can thus be tested. One unresolved methodological issue in TMS experiments is the question of how to adequately calibrate stimulation intensities. The motor threshold (MT) is often taken as a reference for individually adapted stimulation intensities in TMS experiments, even if they do not involve the motor system. The aim of the present study was to evaluate whether it is reasonable to adjust stimulation intensities in each subject to the individual MT if prefrontal regions are stimulated prior to the performance of a cognitive paradigm.

**Methods and Findings:**

Repetitive TMS (rTMS) was applied prior to a working memory task, either at the ‘fixed’ intensity of 40% maximum stimulator output (MSO), or individually adapted at 90% of the subject's MT. Stimulation was applied to a target region in the left posterior middle frontal gyrus (pMFG), as indicated by a functional magnetic resonance imaging (fMRI) localizer acquired beforehand, or to a control site (vertex). Results show that MT predicted the effect size after stimulating subjects with the fixed intensity (i.e., subjects with a low MT showed a greater behavioral effect). Nevertheless, the individual adaptation of intensities did not lead to stable effects.

**Conclusion:**

Therefore, we suggest assessing MT and account for it as a measure for general cortical TMS susceptibility, even if TMS is applied outside the motor domain.

## Introduction

Transcranial magnetic stimulation (TMS) has become increasingly important in brain research and is widely used for noninvasive evaluation and modulation of cortical function. Especially in neuropsychology, TMS gives insight into the function of a circumscribed brain area [1]. The temporary manipulation of neural activity impedes subjects' performance, as measured in prolonged reaction time (RT) and/or higher error rates, even beyond the duration of the stimulation [2]. Behavioral improvements have been reported after the application of certain TMS protocols as well [3]. It therefore allows the establishment of a causal relationship between structure and function. Nevertheless, individuals respond very differently to magnetic stimulation, and it is still unknown how a comparable biological TMS effect can be induced across subjects and how TMS intensity should be ideally gauged. Several factors have been shown to influence subjects' sensitivity to the magnetic pulse like, for example, the individual anatomy with varying distance from the coil to the underlying cortex [4]. Additionally, the trajectories of white matter fibers [5] seem to have an impact on the TMS effect. Moreover, excitability also seems to depend on neuromodulator balance [6] and the functional state of the target area during stimulation [7].

From a technical point of view, the impact of TMS on a cluster of neurons depends on coil type, the geometry and direction of the induced electric field, which is determined by coil orientation [8]–[10], as well as on the pulse waveform defined by the stimulator model [11]. The assessment of these parameters was predominantly performed in the motor cortex; here, the responsiveness of cortical neurons can be measured in an objective way by registering observable muscle twitches or electromyographically monitored motor-evoked potentials (MEPs), often recorded from the first dorsal interosseus (FDI) muscle of the contralateral hand. The stimulator output which is needed to produce a reliable electromyographic response is commonly defined as the motor threshold (MT) [12], [13]. Likewise, cortical excitability can be measured in the visual system by evoking phosphenes [14], brief visual phenomena that are not caused by retinal stimulation. As with the MT, the cortical susceptibility to TMS in the occipital lobe can be determined by measuring the phosphene threshold (PT), with the PT then being the minimum stimulator output necessary for a reliable induction of phosphenes. There are, however, some important shortcomings in the assessment of the PT as compared to MT, because the investigator has to rely on subjective reports, meaning that the effect cannot be measured in an objective way. Additionally, subjects need to be trained to recognize phosphenes and not everyone can perceive them.

In summary, cortical excitability can be measured in both the primary motor and visual cortex. But can regional excitability as expressed in MT and PT be used as a reference for stimulation in non-primary cortical areas? In other words, is there a global excitability level for each individual? Is it therefore valid to calibrate intensities according to those measures if other brain areas are stimulated? In order to elucidate these questions, several studies focused on whether and how MT and PT might correlate. Stewart et al. [15], amongst others [16]–[18], claim that there is no significant correlation between MT and PT. As a consequence, it would not be plausible that the excitability of any other brain region should relate to one of the two thresholds. Hence, adjusting stimulator output according to motor threshold would not reduce the variability of the TMS effect. Under this assumption, a predetermined, invariable intensity across all subjects has been applied in various studies [19]–[26]. On the other hand, one recent study by Deblieck et al. [27] does report a correlation between MT and PT and claimed that methodological shortcomings in the above-mentioned studies might be responsible for the discrepancies. If this was the case, stimulation intensity should be adjusted according to individual MT in order to reduce variability in the effect size, like it has also been practiced previously [28]–[35]. At the moment, the problem of generalizability of thresholds can be regarded as being unsolved and it appears to be more a matter of tradition and intuition whether experimental TMS in other cortical areas is referenced to motor (or phosphene) threshold or not.

Here, we address the question of how to calibrate stimulation intensity to prefrontal areas when performing cognitive experiments, in this case an n-back working memory task. Working memory is frequently defined as a system for the temporary storage and manipulation of remembered information [36]. The well-established n-back task requires this kind of cognitive processing and is known to evoke pronounced activation in the dorsolateral prefrontal cortex (DLPFC) or, more precisely, in the posterior part of the left middle frontal gyrus (pMFG; for a review see[37], [38]). This is in line with cytoarchitectural and functional studies that suggest an important role of the posterior part of the middle frontal gyrus (Brodmann Area 9/46) in working memory processes [39], and due to its location near the cortical surface there is good access to this area using TMS. We located the pMFG as the TMS target region of interest (ROI) in individual functional magnetic resonance imaging (fMRI) maps of 2-back task activation. Because it has been shown that TMS affects local field potentials (LFPs) and BOLD-responses [40], [41], we included a supplementary fMRI scanning session with a subgroup one week after the TMS experiments had ended, as an additional validation of consistency of the localizer, to ensure that the task-related BOLD-response in the target region had not shifted or decreased significantly. The TMS experiments consisted of four separate sessions, in which we applied repetitive TMS (rTMS) with either a predetermined ‘fixed’ intensity, identical for all subjects, or an individually adapted MT-related intensity prior to the working memory task. Both stimulation intensities were also applied to a control region not related to the task (vertex) in order to control for unspecific TMS effects.

If the excitability of the prefrontal cortex relates to the motor system's excitability, we expect most consistent results (i.e., lowest between-subject variability of the TMS-effect) in sessions with individually adapted intensities; at the same time, subjects should show differential effects in response to the pre-determined, fixed intensity, depending on whether they receive a relatively high or low stimulation intensity as compared to their individual sensitivity. In contrast, if there is no relationship between excitabilities, effects should be most pronounced when subjects are stimulated with the highest maximum stimulator output (MSO) (which we apply to the subjects with the highest MT in the individually adapted stimulation sessions); the pre-determined MSO, however, should then result in rather comparable effects across subjects.

## Materials and Methods

### Ethics Statement

All subjects gave their written informed consent, corresponding to the Code of Ethics of the World Medical Association (Declaration of Helsinki, sixth revision in 2008). The study was approved by the ethics committee of the University of Leipzig.

### Subjects

Fifteen healthy subjects (mean age: 24.7 years, SD: 2.8 years) participated in the study. In order to reduce inter-subject variability, we chose only right-handed male participants (handedness was assessed with the Edinbourgh inventory [42]) which are known to be a homogenous and stable group concerning their cortical excitability [43]. They had no history of neurological or psychiatric illness or knew of any cases of epilepsy amongst first-degree relatives. This was assessed with a questionnaire similar to the one established by Keel and others [44].

### Experimental Design

**Figure 1:**
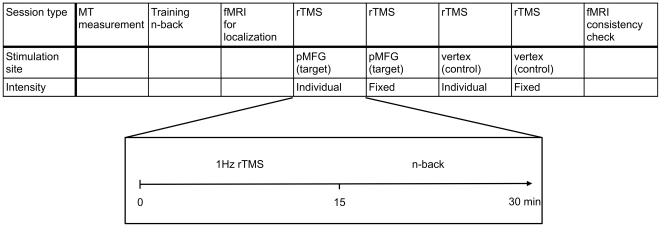
Overview of the experimental procedure. Note that the order of stimulation site and intensity is exemplary for one subject. The second fMRI experiment was only conducted with a subset of the sample (6 out of 15).

All sessions (training, fMRI and four TMS sessions; see Figure 1) were one week apart. In the first week, subjects trained the n-back task in order to reduce training effects during the subsequent sessions. In the second week, the task was performed in an fMRI environment in order to acquire an anatomical and functional localizer for the target region, i.e. the activation peak in the left pMFG. During the next four weeks, we conducted the TMS sessions consisting of off-line stimulation followed by the task.

Overall, subjects were stimulated twice over the target region (pMFG) and twice over the control region (vertex), each time with either a predetermined fixed intensity (i.e., same for all participants) or an individually adapted intensity. The order of stimulation intensity and target site in the four sessions was pseudo-randomized and counterbalanced across participants. To assess stability of the functional localizer after this relatively high number of n-back task runs, a subgroup of six subjects underwent another fMRI scan in order to verify that the activation of the pMFG had not shifted or notably decreased.

### Behavioral Task

**Figure 2:**
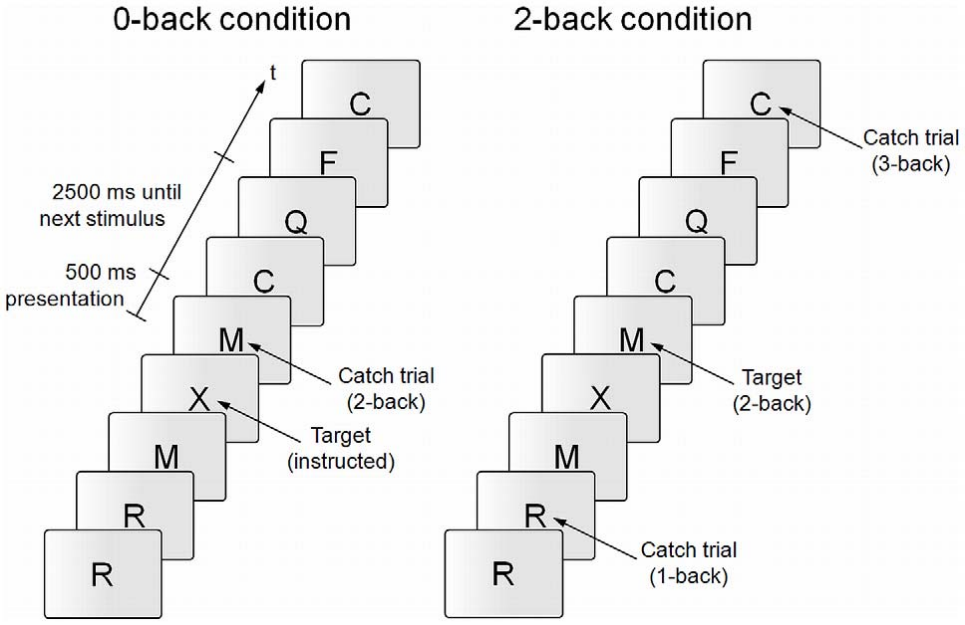
Exemplary extract of experimental block n-back conditions. Participants were instructed to respond to the stimuli labeled as ‘target’ with a right-hand index finger button press.

For the n-back task, a series of letters had to be monitored with the instruction to respond whenever a letter appeared that was identical to the one presented n before (see Figure 2). Usually, n is a predefined integer as 1, 2 or 3. While a 1-back task does not reliably engage working memory, the interpretability of the results of a 3-back task are questioned because the performance decreases extremely and, additionally, there is a supposed capacity constraint [45]. Therefore we used a 2-back condition which still requires constant working memory updating and online-monitoring of the stored information. The so-called 0-back task served as standard experimental baseline condition (e.g., [46]). This is a simple target detection test, which does not need updating of information within working memory because subjects are required to respond whenever a predetermined, i.e., instructed, target letter occurs.

Each experimental block consisted of 25 stimuli out of which six were targets and 19 were non-target letters. In order to control for the possible strategy to simply respond to repeating stimuli in any of the conditions, two of the 19 non-target letters served as catch-trials (two 2-backs in the 0-back condition, one 1-back and one 3-back in the 2-back-condition). Each weekly session consisted of five blocks per condition (presented alternately and randomly beginning with either condition), so that 60 correct responses could optimally be obtained (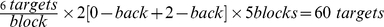). In order to maximize a left lateralization of the activation, we used verbal stimuli (20 consonant letters; cf. [37]). Stimuli occurred in a pseudo-randomized order, with a stimulus duration of 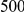 ms at an inter-stimulus interval of 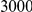 ms. As a measure of accuracy, the discrimination rate P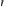 was calculated. P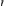 is the difference between hit rate (i.e., correctly detected targets as indicated by a button press) and false alarm rate (i.e., erroneous responses to catch and foil trials), which is a recommended accuracy measure in target recognition tasks like the one described here [47].

### fMRI-Localizer

For individual functional localization, subjects performed the paradigm in a 3T scanner (Medspec S300, Bruker, Ettlingen, Germany). Twenty axial slices (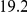 cm field of view, 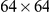 matrix, 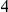 mm thickness, 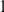 mm spacing) parallel to the AC-PC plane and covering the whole brain were acquired using a single shot, gradient recalled echo-planar imaging (EPI) sequence (TR 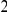 s, TE 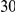 ms, 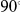 flip angle).

#### Preprocessing and Co-Registration

Data processing was performed using the software package LIPSIA [48], which contains tools for preprocessing, co-registration, statistical evaluation, and visualization of fMRI data. Distortion correction was performed using a fieldmap scan that was acquired prior to the functional scan. Data were then corrected for motion using a matching metric, based on linear correlation. To correct for the temporal offset between the slices acquired in one scan, a cubic-spline-interpolation was applied.

To align the functional data slices with a 3D stereotactic coordinate reference system, a rigid linear registration with six degrees of freedom (three rotational, three translational) was performed. The rotational and translational parameters were determined on an optimal match between modified driven equilibrium Fourier transform (MDEFT; [49], [50]), EPI-T1 scans that were run prior the functional data acquisition and an individual 3D reference data set, which had been measured for each subject during a previous scanning session. The high-resolution 3D reference data set with 160 slices and 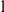 mm slice thickness was standardized to the Talairach stereotactic space [51]. The rotational and translational parameters were subsequently transformed by linear scaling to standard space. The resulting parameters were then used to transform the functional slices using trilinear interpolation, so that the resulting functional slices were aligned with the stereotactic coordinate system, and functional data was re-sampled to 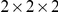 mm. A temporal high-pass filter with a cut-off frequency at 1/100 Hz was used for baseline correction of the signal and a 3D spatial smoothing was applied with a Gaussian filter of 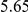 mm full width at half maximum (FWHM), which is equivalent to 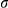 = 1.2.

#### Statistical Analysis

The statistical evaluation was based on a least-squares estimation using the general linear model for serially auto-correlated observations [52]–[55]. Data were modeled as mixed design including block-related information about the 0-back and 2-back conditions as well as pause/instruction periods between these task blocks, and event-related information about correctly detected 0-back and 2-back trials. The design matrix was generated with a synthetic hemodynamic response function [56], [57] and its first derivative (for events only). The model equation, including the observation data, the design matrix and the error term, was convolved with a Gaussian kernel of dispersion of 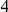 s FWHM to deal with the temporal auto-correlation [55].

#### ROI Definition

In order to optimally determine the individual localization of the region of interest (ROI), i.e., the target of stimulation, 2-back vs. 0-back block-related activity were contrasted resulting in z-maps indicating activation differences between these conditions for each subject. The identification of the target region was based on functional and anatomical criteria (i.e., the highest z-value (Z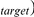 within the pMFG of the individual brain was classified as the ROI; see Table 1). For these single-subject data, no threshold or cluster size criteria were applied.

**Table 1:** Coordinates and Z value maximum of individual pMFG target regions.

	Coordinates (mm)			
Subject	x	y	z	Z 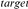
1	−34	8	38	1.61
2	−35	5	47	2.03
3	−35	4	52	2.09
4	−27	1	44	3.10
5	−45	33	38	1.60
6	−40	0	35	2.13
7	−46	38	17	3.21
8	−52	−1	34	3.41
9	−37	16	42	1.52
10	−36	−6	33	2.40
11	−34	13	37	1.32
12	−47	−4	44	1.99
13	−31	−2	55	1.96
14	−38	12	37	2.96
15	−38	14	32	2.33

Note that images were not normalized prior to fitting to the Talairach coordinate system provided by Brainsight; compatibility between subjects is therefore limited. Reference point for the origin of the underlying coordinate system is the anterior commissure (AC: 0 0 0) with the axial plane through anterior and posterior commissures (AC-PC) and the z-axis orthogonal to this plane. Values are like in the standard coordinate systems in mm with −x - left, +x - right, −y - posterior, +y - anterior, −z - inferior, +z - superior.

However, in order to compare our fMRI activation patterns with the existing ones in the n-back literature (for a review see: [37]), average statistical parametric maps based on the individual contrast images of the 2-back vs. the 0-back condition were computed.

The individual z-maps were converted to NIfTI-format and transferred to a PowerMac G5 (Apple Inc., Cupertino, USA) and used for localization with Brainsight TMS Software Version 1.7.8 (Rogue Research Inc., Montreal, Canada). The connected infrared stereo camera (Polaris Optical Tracking System, Northern Digital Inc., Waterloo, Canada) can detect both the coil and the participant by means of attached reflective trackers. This procedure provides the best precision in coil guidance [58] and furthermore allows reproduction of trajectories (i.e., the exact position and angle of the coil) in repeated sessions.

### TMS

#### Threshold Measurement

Stimulation was performed with a biphasic magstim Rapid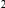 stimulator (The MAGSTIM Company Ltd, Whitland, UK) and a 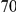 mm figure-of-eight-shaped coil. Motor-threshold assessment followed recommendations by Rossini and Rothwell [12], [13]: The coil was held tangentially to the skull at a 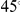 angle to the corresponding parasagittal line. MEPs were recorded from the right FDI using self-adhesive Ag-AgCl gel electrodes in a standard belly-tendon fashion. A Digitimer 360 (Digitimer Limited, Welwyn Garden City, UK) amplifier and the Spike Software Package version 4.02 (Cambridge Electronic Design Ltd, Cambridge, UK) were used to detect electromyographic responses. In order to find the MT hot-spot, we started with 55% MSO, which was sufficient in most participants to evoke MEPs; otherwise, intensity was increased in 5% steps. We started the stimulation 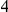 cm lateral and 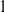 cm anterior to the vertex. We systematically shifted the coil in steps of approximately 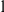 cm in anterior, posterior, lateral and medial direction, respectively, and repeated repositioning if higher MEPs were elicited from the new position. If coil replacement did not result in higher MEPs, we reduced the power at first in two then in one percent MSO decrements.

MT was both quantified at rest (rMT) as well as during activation (aMT). The rMT was defined at the percentage of MSO needed to evoke 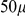
V MEPs peak to peak in five out of ten consecutive trials. The aMT was assessed while the subjects had to contract the FDI with 20% of maximum force, which was measured with an analog dynamometer. The aMT was defined as the minimum MSO that would elicit MEPs exceeding contraction activity by 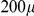
V in at least five out of ten consecutive stimulations. The MTs were measured in independent sessions prior to the actual experiment.

#### rTMS

Using an air-cooled figure-of-eight coil, we applied a continuous 1 Hz rTMS stimulation protocol for 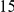 min (total of 900 pulses) prior to task performance. The coil was held tangentially to the skull with the handle pointing upwards, and position was adjusted so that the electric current in the center of the coil would run perpendicular to the course of the inferior frontal sulcus [9], [59]. As control site, the vertex was stimulated with the induced current running from posterior to anterior along the interhemispheric fissure. Stimulation intensity was adjusted to 90% of the individual rMT or set at a fixed intensity of 40% MSO. Ideally a fixed intensity should be set to the group average MT. However, the fixed intensity was chosen at 40% MSO because it corresponded to the rMT of the subject with the lowest thresholds and could thus be considered safe for all participants [60].

#### Scalp-Cortex Distance Measurement

MT has been shown to be influenced by the distance between the scalp and the cortex within the primary motor cotrex (M1). Scalp-cortex distance (SCD) has been claimed to account for 25% [61], 29% [62] or even 45% [4] of interindividual MT variance. Therefore, we measured the SCD using the built-in Brainsight TMS Software metering rule in the target stimulation area and M1. In the high-resolution 3D-data set, we measured the distance from the closest voxel within the scalp surface to the target cortical site (pMFG) in all three axes in mm [62]. As we did not use neuronavigation in MT assessment, we measured the SCD in the neuroanatomical location of the hand-area (M1) above the hand-knob.

## Results

### Motor Threshold

rMT varied between 40 and 72% of MSO (mean rMT = 52.8; SD = 9.4), whereas aMT showed a variance between 37 and 68% of MSO (mean rMT = 47.4; SD = 8.0) (see Table 2). A two-tailed Pearson-test revealed a significant correlation of rMT and aMT (r = 0.893; p
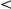
0.01).

**Table 2:** Motor thresholds and SCD.

			SCD in mm	
Subject	aMT	rMT	pMFG	M1
1	58	67	9	12.1
2	45	48	13.1	17.3
3	50	50	16.3	18.9
4	40	44	9.7	10
5	43	48	12.2	11.4
6	45	53	8.9	10.7
7	54	70	11.3	14.2
8	51	55	14	18.5
9	37	49	9	14.1
10	42	44	10	11.6
11	39	40	9.5	11.9
12	47	50	14.3	15
13	48	50	11.3	13.6
14	44	48	10.5	19.1
15	68	72	14.6	18.6

Motor threshold is expressed as % of MSO; note that SCD in pMFG was measured at the target stimulation site and the SCD in the M1 was measured on the anatomical area of the representation of the hand (handknob).

### Scalp-Cortex Distances

A one-tailed Pearson's correlation showed that SCD in the pMFG and the hand-knob did correlate (r = 0.717; p
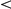
0.05). In M1, a one-tailed Pearson's correlation of SCD with aMT revealed a non-significant trend (r = 0.431; p
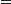
0.055), whereas rMT did not show any significant correlation with the SCD in the hand area (r = 0.264; p
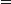
0.171) (see Table 2).

### fMRI Localizer

In the fMRI-contrasts (2-back vs. 0-back), we found activation in the posterior part of the left middle frontal gyrus (pMFG) in all participants. In the single subject contrasts, the z-value-threshold was gradually lowered until one blob within the pMFG with only one local maximum could be identified unambiguously. The peak voxel of this blob was then marked as the target stimulation site. Activation strength of the target, as indicated by the z-value of the local maximum (Z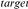), varied between subjects (range: 1.32–3.41; mean 2.24; see Table 1) and was therefore included as regressor in later analysis in order to rule out an influence on effects of the stimulation.

**Figure 3:**
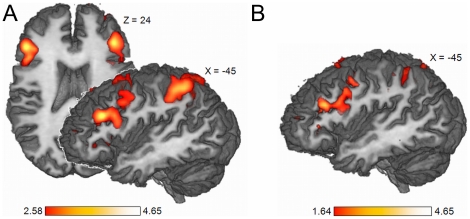
fMRI Results. (**A**) Averaged (n = 15) block-related 2-back vs. 0-back activity resulting in pMFG activation (peak voxel Talairach coordinates −45 26 24). Displayed are only voxels with z
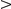
2.58 (i.e., p
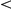
0.005). (**B**) fMRI check for consistency. Conjunction of averaged (n  = 6) 2-back vs. 0-back block-related activity derived from pre- and post-TMS scans (peak voxel Talairach coordinates −45 26 24).

The averaged contrast images of the 2-back vs. the 0-back condition revealed significant activation in the pMFG (Peak voxel Talairach coordinates −45 26 24, threshold at z
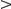
2.58, i.e., p
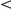
0.005, see Figure 3A). For the subsample of six participants undergoing a second post-TMS fMRI scanning session, the same average z-map was computed. Subsequently, a conjunction analysis of these subjects' pre and post TMS 2-back related activity was conducted, resulting in the same activation pattern in the pMFG (see Figure 3B).

### Behavioral Data

#### General Effects

**Figure 4:**
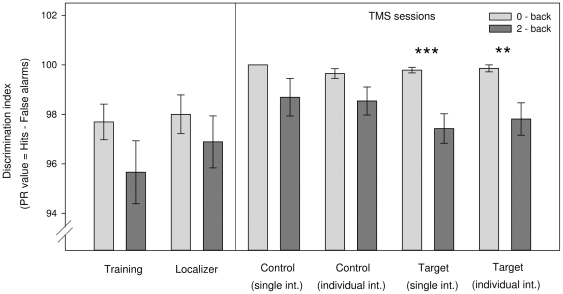
General effects. Task performance on the two experimental conditions (2-back vs. 0-back) as measured by P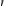. Paired-sample t-tests only revealed a significant difference when subjects were stimulated in the target region both with a single intensity (single int.: P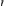 = 99.8 vs. 97.4; t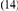 = 4.150, p
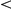
0.001) and with an individually adapted intensity (individual int.: P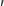 = 99.9 vs. 97.8; t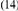 = 2.989 p
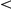
0.01).

Calculating paired sample t-tests (2-back vs. 0-back), we found a significantly different performance (P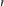-values) after stimulating the DLPFC both with a single intensity (P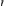 = 99.8 vs. 97.4; t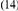 = 4.150, p
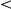
0.001) and with an individual intensity (P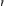 = 99.9 vs. 97.8; t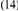 = 2.989 p
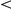
0.01). Performance in the 2-back task did not differ significantly from 0-back performance in the training or scanning sessions (see Figure 4).

The mean RT included only trials that were defined as targets (i.e., 0- and 2-back trials) in the respective block and were detected correctly by subjects. There was a significant difference between the two conditions, indicating slower RTs on 2-back trials (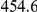 ms vs. 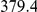 ms; t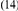 = 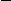
2.812, p
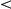
0.05) in the very first session (i.e., the training session). All other sessions did not show any significant difference in RTs between conditions.

#### TMS-Specific Results

A 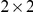 repeated measures ANOVA with the factors stimulation site (pMFG vs. vertex) and n-back condition (2-back vs. 0-back) was calculated separately for the sessions with a single intensity and the sessions with an individual intensity. Stimulating with a single intensity did not show any systematic effects, i.e. P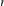 and RT did not differ significantly between the target and control sessions.

**Figure 5:**
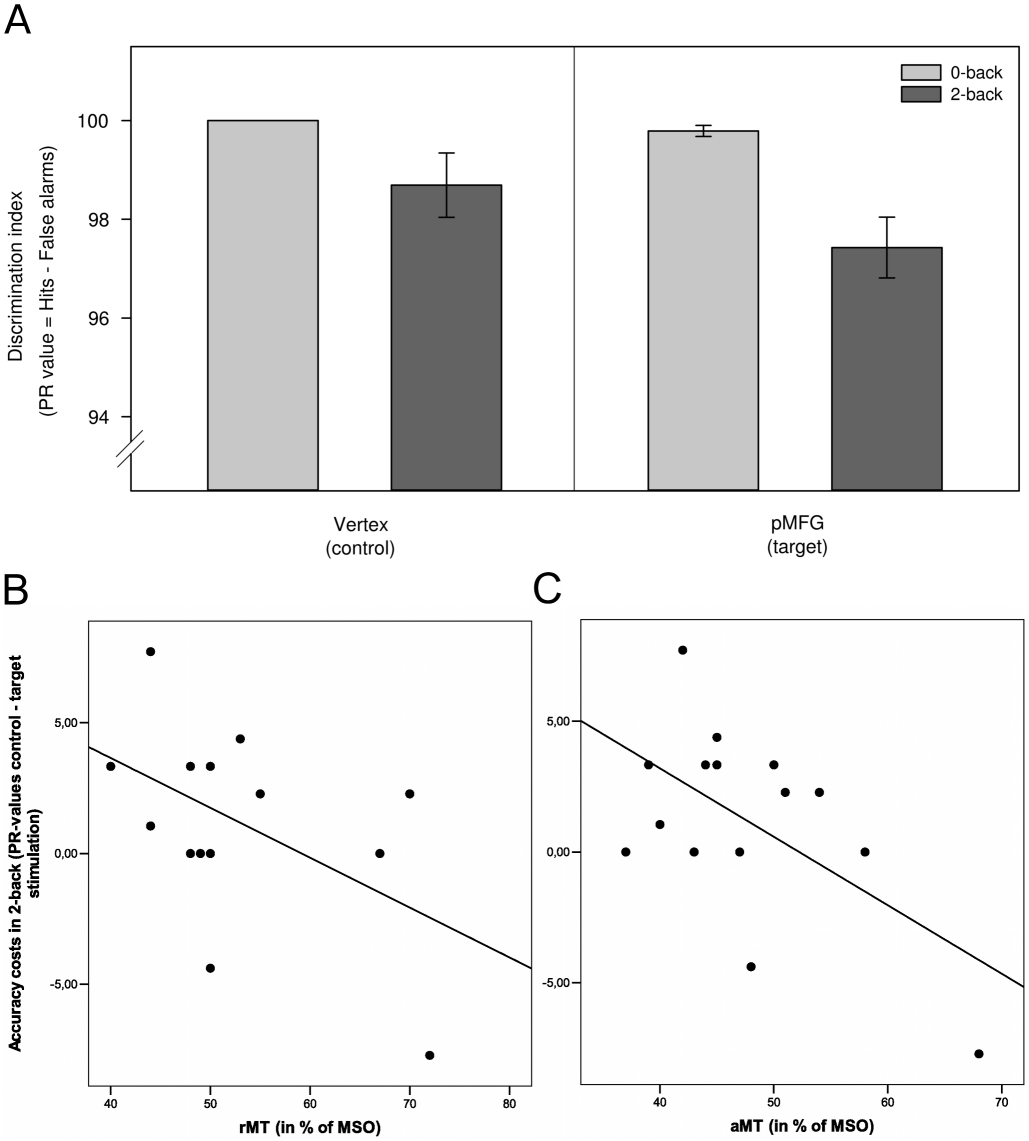
TMS-specific results. (**A**) Interaction between stimulation site and block type after stimulation with a fixed intensity (40% MSO). A sufficient level of statistical significance of that interaction was only reached when the repeated measures ANOVA was informed with the rMT as a covariate (F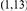 = 5.311, p
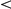
0.05). (**B**) Correlation of resting motor threshold and costs in task accuracy, as revealed by the difference between control and target stimulation P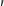 value (rMT: 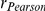 = 0.488; p
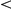
0.05). (**C**) Correlation of active motor threshold and costs in task accuracy, as revealed by the difference between control and target stimulation P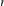 value (aMT: 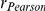 = 0.487; p
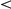
0.05).

In order to test whether the fixed intensity had differentiated effects on participants' performance based on their individual MT, we introduced a covariate which expresses 40% MSO as a percentage of individual MT (
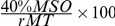
). As a consequence, we found a significant interaction between stimulation site and n-back condition (F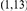 = 5.311, p
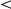
0.05) concerning task performance (P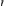-values; see Figure 5A). In a subsequent post-hoc analysis, we correlated the rMT with the decrease in task attainment. The rMT correlated with 2-back performance (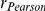 = 0.488; p
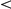
0.05), indicating that a lower rMT lead to a larger decrease in task performance (see Figure 5B), whereas the 0-back condition did not show a significant correlation.

As rMT and aMT were highly correlated, we performed the same analysis with aMT as covariate. Again, the ANOVA revealed a significant interaction between stimulation site and n-back condition (F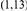 = 5.004, p
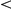
0.05), with a corresponding correlation (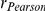 = 0.487; p
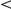
0.05; see Figure 5C).

**Figure 6:**
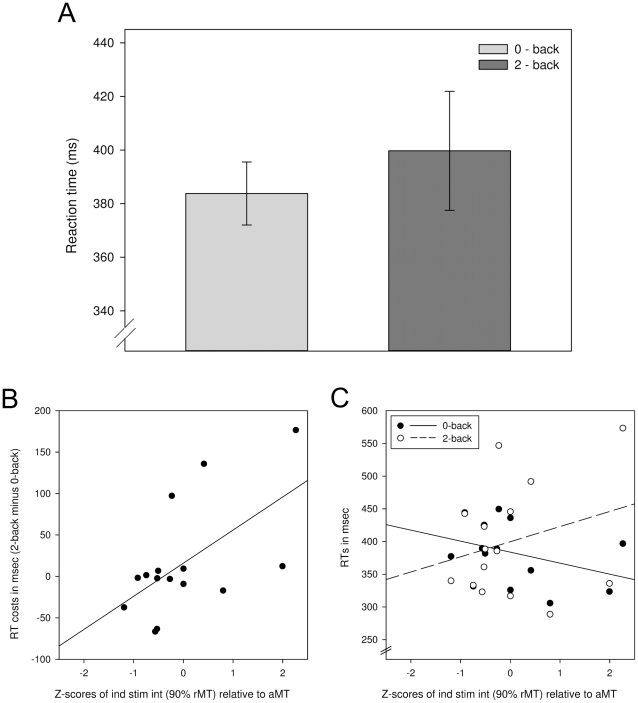
Non-specific influence of rTMS on task performance. (**A**) Statistically significant difference between 2-back and 0-back condition after stimulation with individually adapted intensity when informing the model with the aMT as a covariate, or the z-score of percent rMT in relation to aMT, respectively (F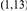 = 6.981, p
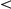
0.05) (**B**) Correlation analysis indicating higher RT costs (i.e., 2-back minus 0-back RT) correlating with higher stimulation intensity in relation to aMT (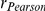 = 0.583; p
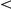
0.05) (**C**) The higher RT costs could not be attributed to an increase in 2-back RTs or decrease in 0-back RTs alone, but rather a combination of both.

When calculating the 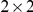 ANOVA for the individually adapted stimulation intensity, no significant interaction was found. However, to test the possibility that aMT might be the better reference for overall excitability, we recalculated post-hoc the individual stimulation intensity applied (90% rMT), so it would be expressed as a percentage of aMT (
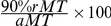
). As a consequence, we found a non-TMS-specific effect in the RT difference between 2- and 0-back condition (F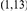 = 6.981, p
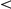
0.05) (see Figure 6A). The decrease in RT n-back effect correlated with the stimulation intensity relative to the aMT ( = 
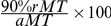
) (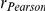 = 0.583; p
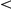
0.05) (see Figure 6B). Further analysis revealed that neither the 0-back nor the 2-back block alone accounted for this, but rather the interaction between an increase in 2-back RTs and a decrease of 0-back RTs (see Figure 6C). Neither SCD nor Z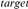 explained any variance when included as covariates to the ANOVA.

## Discussion

### The Problem

When applied in non-motor regions for neuropsychological studies, the calibration of intensity is one of the major problems in TMS studies [63]. This is due to the high interindividual variance in cortical sensitivity and the uncertain intraindividual transferability of measurable thresholds. Ideally, the effect of the stimulation should be comparable between subjects. In order to achieve small intersubject variance, individually adapted stimulation intensity is desirable. Some researchers try to achieve this by adapting the stimulation intensities according to the MT. There are, however, good reasons to distrust MT as a valid measure for the sensitivity in non-motor cortical regions. As for any two given target areas, the cytoarchitectue of the cortex, the gyrification, the hodological structure, and the anatomy of the skull are different in prefrontal areas and the primary motor cortex. Therefore, the effect of TMS and the sensitivity to stimulation might be very different.

### Stimulation Intensities

Our results demonstrate that there is a link between cortical TMS excitability in the two regions examined (motor and prefrontal cortex). When all participants were stimulated with the same intensity (40% MSO), changes in performance (P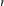) related to rMT: impact was strongest on subjects with a lower rMT (e.g., 40% MSO), who were stimulated at comparatively high intensities (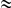
100% rMT); in contrast, for subjects with a high rMT (e.g., 72% MSO), the 40% MSO fixed intensity stimulation was far below their threshold and did not have a significant effect (see Figure 5B). Absolute stimulation intensity (in % MSO) alone had no significant influence either; otherwise, subjects with higher thresholds should have shown greater deterioration in the sessions with individually adapted stimulation, because here they received the strongest stimulation of all subjects. However, if excitability of the non-motor cortices is related to rMT, why did individually adapted stimulation at 90% rMT yield no significant effects?

The most plausible explanation is that 90% rMT is not the most effective intensity for the present target site within the prefrontal cortex. Possibly, higher threshold-adapted intensities would yield stronger effects. However, it is known that higher stimulation intensities are not automatically more efficient [64] and the DLPFC might have a much higher or lower (TMS-)sensitivity than the motor cortex. Both an over- and an under-stimulation of any cortical area might lead to no, different, or opposite effects, depending on the neuronal populations predominately activated at any point. In principle, there might be any kind of non-linear yet stable relation between MT and the “DLPFC threshold”. Therefore, we cannot tell how to ideally adjust stimulation intensity. Based on our findings, we suggest that MT serves as a suitable measure of varying excitability between individuals. However, it does not provide an easy-to-use reference of how to stimulate any other part of cortex within the same brain, especially if basic features (cortical cytoarchitecture, etc.) differ.

After correcting individual stimulation intensity for aMT (
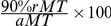
), we found an impact of stimulation on response times over the whole period of the task, but surprisingly the effect was not specific for the stimulation site (pMFG vs. vertex). This indicates that the control stimulation to the vertex might not be the ideal option and possibly stimulates some midline cortical areas as the supplementary motor area, which could interfere with context-dependent motor planning. Choosing an adequate control site is yet another methodological issue that may be addressed in different ways: Given that stimulation to the DLPFC can result in disturbing jaw and facial muscle twitches, control stimulation should ideally elicit similar side effects, which is not true for either sham (tilted coil) or vertex stimulation [65]. Therefore, contralateral stimulation might be the best choice with regards to side-effects. But in the given working memory task, lateralization is often only partial, i.e., the contralateral site is involved in a cognitive process, and contralateral stimulation might have effects of its own. Therefore, we think that vertex stimulation is still the better choice, eliciting local somatosensory skin sensations, albeit not comparable to target stimulation.

### Recommendation Based on Our Results

One of our main results is that there was an influence of individual MT on stimulation effects when all participants were stimulated at the same MSO, as we could show by a regression analysis. However, individually adapted stimulation yielded no significant TMS effects.

Based on these findings, it is not possible to say how the optimum DLPFC stimulation intensity would have to be adjusted. Therefore a whole range of intensities would have to be tested in order to evaluate a possible threshold in a specific brain area, which requires extremely meticulous and tedious studies, especially when involving cognitive processes. In most experiments, this would not be feasible if the determination of the threshold is not the overall aim of the study. Thus we suggest to use a fixed stimulation intensity across subjects, but include the individual MT in regression analyses, as an estimate of between-subject variability in cortical excitability. This idea would be in line with Deblieck et al. [27] who also suggested an intersubject comparability of different cortex areas concerning TMS excitability. Additionally, we could show that neither SCD nor differences in the fMRI data, depicted as Z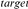, had a significant impact on the measured TMS-Effects (i.e., change in P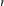-value). Hence, we conclude that MT is the best marker for interindividual differences in TMS susceptibility.

### TMS-Limitations

In any TMS experiment, one should consider sources of variance lying in the method itself: as discussed above TMS efficacy might be modulated to a variable degree by to different SCDs [61]. The fact that SCD in M1 and prefrontal regions correlate can be corrected e.g., by algorithms as the one introduced by Stokes et al. [62]. The adjustment of stimulation proposed in their publication, however, cannot take differences in tissue composition and conductivity into account [63]. In our subset of participants, we found a nonsignificant trend for the correlation of aMT and SCD in M1. However, as mentioned above, this finding could only partially explain a relationship between those two areas. The finding that rMT did not correlate with SCD in M1 might be due to the fact that rMT is not as precise as aMT as a measure of excitability.

Secondly, the induced effect is supposed to be a circumscribed cortical disturbance leading to measurable behavioral changes. It is, however, known that whole functional networks can be affected [25], [66]. Possible long-distance effects are often ignored and some networks might prove functionally more resistant to TMS than others. Another interesting point to consider is the state dependency of TMS [7]. Adapted to our experimental design, one could speculate that any random cognitive operation performed by the subject during the stimulation period might possibly have an impact on stimulation efficiency, and thus subsequent behavioral changes, due to a different state of neuronal excitation.

Another finding noteworthy to be pointed out is the effect that rhythmic TMS patterns like the one applied here can have an effect on brain oscillations, some of which have been linked to cognitive tasks (for a review, see [67]). Although we cannot rule out that our TMS protocol caused such an entrainment of natural oscillations [68], we do not have to assume that it influenced the results. While the oscillations have been related to various TMS frequencies, regardless of intensity, we compared different intensities at the same frequency.

Concerning effects of the repetitive performance of the n-back task, a conjunction analysis was conducted for a subsample (i.e., n = 6) for their pre- and post-TMS fMRI scans. We found precisely the same activation pattern in the pMFG that was evident in the averaged pre-TMS fMRI scans of the whole sample (i.e., n = 15). Hence, we conclude that neither the excessive performance of the n-back task nor the repeated application of rTMS over a period of four weeks result in notably weaker recruitment of the same cortical network or the use of entirely different brain regions. Therefore, we may interpret all of the TMS effects as being exclusively attributable to the manipulation of stimulation intensity and site.

### Conclusion

We found a link between cortical sensitivity in the motor cortex, and the left dorsolateral prefrontal cortex. However, the exact magnitude of this correlation remains unclear. As long as there is no strong hypothesis concerning the absolute excitability of the DLPFC, we suggest stimulating all subjects with the same stimulation intensity, and subsequently correcting for individual thresholds in a regression analysis. We also believe that this procedure is feasible for other non-primary cortical areas than the DLPFC. However, future research is necessary to prove if this is actually true.

Additionally, we showed the stability of an n-back fMRI-localizer over a total of six sessions, two of them including TMS to the peak of activation in the left pMFG; this consistency, which is crucial to any experiment including fMRI and TMS in different sessions, is implicitly assumed but, as to our knowledge, hadn't been shown previously.
